# The Illinois State Dental Society

**Published:** 1886-07

**Authors:** 


### THE ILLINOIS STATE DENTAL SOCIETY.

It is seldom that so much matter of vital import to dentistry is offered in such small compass as may be found in the remarks of Dr. Black, in the opening pages of the report in this number. There is no chance for misapprehension, for he is so clear and lucid in his statements, so comprehensive and yet so concise in language, that the wayfaring dentist, though a fool, may not err in comprehension. There has been so much of misunderstanding concerning the nature and action of bacteria in the mouth, that every one should carefully study these presentations. It is positively nauseating to hear the crude, mistaken interpretations of the latest teachings of science, that are sometimes enunciated in society meetings by otherwise intelligent men. There is no excuse for this ignorance when such clear presentations as those of Miller and Black are to be found in the journals.

